# Association between Air Pollution and the Development of Rheumatic Disease: A Systematic Review

**DOI:** 10.1155/2016/5356307

**Published:** 2016-10-25

**Authors:** Gavin Sun, Glen Hazlewood, Sasha Bernatsky, Gilaad G. Kaplan, Bertus Eksteen, Cheryl Barnabe

**Affiliations:** ^1^Department of Medicine, Cumming School of Medicine, University of Calgary, Calgary, AB, Canada; ^2^McGill University, Montréal, QC, Canada; ^3^Department of Community Health Sciences, Cumming School of Medicine, University of Calgary, Calgary, AB, Canada

## Abstract

*Objective*. Environmental risk factors, such as air pollution, have been studied in relation to the risk of development of rheumatic diseases. We performed a systematic literature review to summarize the existing knowledge.* Methods*. MEDLINE (1946 to September 2016) and EMBASE (1980 to 2016, week 37) databases were searched using MeSH terms and keywords to identify cohort, case-control, and case cross-over studies reporting risk estimates for the development of select rheumatic diseases in relation to exposure of measured air pollutants (*n* = 8). We extracted information on the population sample and study period, method of case and exposure determination, and the estimate of association.* Results*. There was no consistent evidence of an increased risk for the development of rheumatoid arthritis (RA) with exposure to NO_2_, SO_2_, PM_2.5_, or PM_10_. Case-control studies in systemic autoimmune rheumatic diseases (SARDs) indicated higher odds of diagnosis with increasing PM_2.5_ exposure, as well as an increased relative risk for juvenile idiopathic arthritis (JIA) in American children <5.5 years of age. There was no association with SARDs and NO_2_ exposure.* Conclusion*. There is evidence for a possible association between air pollutant exposures and the development of SARDs and JIA, but relationships with other rheumatic diseases are less clear.

## 1. Introduction

Environmental exposures and genetic predisposition are hypothesized to interact to result in the expression of autoimmune rheumatic diseases such as rheumatoid arthritis (RA), juvenile idiopathic arthritis (JIA), and systematic autoimmune rheumatic diseases (SARDs) [1], as well as other immune-mediated diseases such as inflammatory bowel disease [2] and multiple sclerosis [3]. Identifying modifiable risk factors for disease development and prognosis is important to reduce the substantial impact and burden of these chronic diseases in society.

Air pollution is a plausible risk factor for autoimmune disease development. Other inhalants such as tobacco smoke and silica are strongly associated with the development of RA, related to their ability to directly interact with alveolar tissue [4, 5]. Air pollution has been demonstrated to be able to directly stimulate an inflammatory response [6] and indirectly alter the microbiome [7]. A relationship between particulate matter exposure and elevations in inflammatory marker levels has been described [8–11]. As randomized controlled trials to assess directly for causation between air pollutant exposures and disease development in humans are not feasible, we must rely on observational studies to assess for evidence of associations. Fortunately, several methods to estimate air pollutant exposure exist. A variety of air pollutants from industrial and private sources are measurable at fixed-site continuous monitoring stations that collect hourly mean levels of criteria air pollutants, including particulate matter <2.5 *μ*m in size (PM_2.5_), particulate matter <10 *μ*m in size (PM_10_), sulfur dioxide (SO_2_), nitrogen dioxide (NO_2_), carbon monoxide (CO), and ozone (O_3_). The hourly data can then be averaged to obtain defined temporal estimates for the region (e.g., a city). Land use regression models use Geographic Information System (GIS) to estimate air pollutant exposure through a combination of land use, traffic, population density, physical geography, and meteorology across an area [12] and predict concentrations at a defined site, such as an individual's location of residence determined by postal code [13]. Inverse distance weighting (IDW) uses the general principle of interpolation, where values at a site are estimated based on distance from a measured value at another point (e.g., a monitoring station) under the presumption of reducing pollutant levels with increasing distance [14]. Finally, remote sensing through satellite imaging yields estimates across broad geographic areas.

Our objective was to identify studies estimating associations between exposure to the air pollutants listed above and the risk of development of select rheumatic diseases. This included inflammatory arthritis conditions such as RA and JIA, as well as SARDs and individual diseases of autoimmune myositis, systemic lupus erythematosus (SLE), scleroderma, and vasculitis.

## 2. Methods

### 2.1. Search Strategy

MEDLINE (1946 to September 2016) and EMBASE (1980 to 2016, week 37) databases were searched using MeSH terms and keywords for rheumatic diseases (RA, SLE, JIA, inflammatory myositis, scleroderma, vasculitis, and SARDs) in relation to exposure to measured air pollutants [15] (Search Strategy in Appendix).

### 2.2. Study Selection

Three authors (Gavin Sun, Glen Hazlewood, and Cheryl Barnabe) independently completed title and abstract and full-text reviews. Studies were included based on the following criteria: assessing the outcome of a rheumatic disease of interest (RA, JIA, SARDs, and individual diseases of autoimmune myositis, SLE, scleroderma, and vasculitis), individual exposure to ambient air pollutants (PM_2.5_, PM_10_, SO_2_, NO_2_, CO, and O_3_), and having a case-control, case cross-over, or cohort design. Only English language studies were included. The study had to report risk estimates (any of relative risk (RR), hazard ratio (HR), or odds ratio (OR)) with the corresponding 95% confidence intervals (95% CI) or sufficient data for calculation. Reviews, case reports, mechanism studies, and nonhuman studies were excluded.

### 2.3. Data Extraction and Assessment of Study Quality

Data extraction was performed in duplicate by two authors (Gavin Sun and Cheryl Barnabe). A standard reporting form was developed to extract pertinent information from each study, including the country or region of study, calendar years of study, diagnosis criteria for the rheumatic disease assessed, and the number of patients in case or control groups in each category. The study design and method of assessing air pollutant levels were also extracted. The estimates and their margin of error were extracted. The Newcastle-Ottawa scale [16] was used to assess the quality of the studies relevant to the objective, again in duplicate by two authors (Gavin Sun and Cheryl Barnabe). For case-control studies, quality was assessed for four domains of selection (case definition, representativeness of cases, selection of controls, and definition of controls), two domains of comparability (study controls for the most important factor and any additional important factor), and three domains of exposure (ascertainment of exposure, same method of ascertainment for cases and controls, and the nonresponse rate). For cohort studies, quality is assessed for four domains of selection (representativeness of exposed cohort, selection of the nonexposed cohort, ascertainment of exposure, and demonstration that the outcome of interest was not present at start of study), two domains of comparability (study controls for the most important factor and any additional important factor), and three domains of outcome (method of assessment of outcome, follow-up period, and adequacy of follow-up of cohorts). Points are assigned based on specified levels of quality within each domain to a maximum of 9 points.

### 2.4. Statistical Analysis

Our a priori study protocol intention was to perform meta-analysis on eligible studies. Following the full-text review stage, we determined that pooling was not appropriate given the small number of studies and heterogeneity in methods; thus the studies were summarized qualitatively.

## 3. Results

### 3.1. Study Inclusion

**Figure 1:**
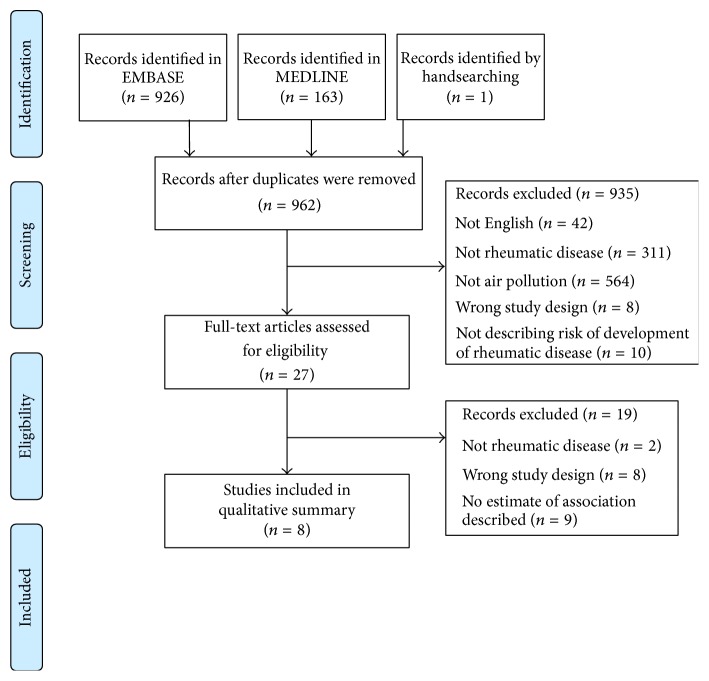
Study selection.

A total of 962 unique publications were identified, of which 27 underwent full-text review, with 8 studies included in our summary [17–24] (Figure 1). Individual study characteristics are listed in Table 1.

We identified studies in RA (*n* = 4), SARDs (*n* = 2), and JIA (*n* = 2) populations; no studies were found for SLE, inflammatory myopathies, or scleroderma as unique entities. With the exception of studies from Sweden and Taiwan, all studies were of North American populations. One abstract each in the conditions of ANCA vasculitis [25] and Kawasaki Disease [26] was found, but they did not report risk estimates and thus were not included in the formal synthesis.

### 3.2. Rheumatoid Arthritis

Four studies included subjects with RA (two case-control studies [18, 19] and two cohort studies [17, 20]) and examined associations with exposure to NO_2_, SO_2_, PM_2.5_, and PM_10_ (Table 2). In Hart et al., 2013, using data from the Nurses' Health Study and land use regression models, there was no definite evidence for increased RA risk related to a cumulative average exposure to NO_2_, SO_2_, PM_10_, or PM_2.5_ after adjustment for covariates [20]. In Hart et al., 2013, using data from the Swedish Epidemiological Investigation of Rheumatoid Arthritis study and land use regression models, the investigators were unable to demonstrate any increased risk for the development of RA with exposure to NO_2_, PM_10_, or SO_2_ [19]. In the study by De Roos et al., RA definitions were based on physician billing and prescription data; land use regression was used in the estimates for PM_2.5_ and NO_2_ as well as additional pollutants, black carbon and NO, and the inverse distance weighting method was used for PM_10_ and SO_2_ estimates as well as for NO, ozone, and CO [18]. When the RA definition required a specialist-confirmed diagnosis, air pollutant exposure effect estimates were all inversely associated with the development of RA. In this study, residence proximity to roadway was additionally studied as a proxy for air pollutant exposure, with a significantly higher risk for RA for those within 50 metres from a highway compared to those over 150 metres away (OR: 1.37; 95% CI: 1.11 to 1.68). In the study by Chang et al., data from monitoring sites were linked to administrative health data and incident RA cases were studied [17]. No association was found for PM_2.5_ exposure, but a significantly higher risk of incident RA was found in those exposed to the highest NO_2_ levels (adjusted HR for 3rd quartile: 1.53; 95% CI: 1.12 to 2.09; adjusted HR for 4th quartile: 1.52; 95% CI: 1.11 to 2.08).

### 3.3. Systemic Autoimmune Rheumatic Diseases

 Bernatsky et al. reported the association between PM_2.5_ exposure and the odds of prevalent SARDs in case-control studies performed in Quebec and Alberta, Canada [21]. Exposure measurement was determined using average residential exposures at diagnosis based on satellite-derived data. In Alberta, a nonlinear association was found. The OR at PM_2.5_ exposures of 6.02 to 6.92 *μ*g/m^3^ was 1.25 (95% Credible Interval (CrI): 1.15 to 1.36), the OR at exposures of 6.92 to 8.11 *μ*g/m^3^ was 1.03 (95% CrI: 0.94 to 1.13), and the OR at exposures of ≥8.12 *μ*g/m^3^ was 1.13 (95% CrI: 1.02 to 1.25) after adjustment for sex, age, urban versus rural residence, and median income. In Quebec, increasing odds for increasing levels of PM_2.5_ exposure were demonstrated, with significant odds at levels of ≥11.81 *μ*g/m^3^. In a study focused on one city in Alberta (Calgary) using land use regression models, exposure to PM_2.5_ appeared to be potentially associated with prevalent SARD (OR: 1.10; 95% CrI: 1.01 to 1.22) in the model adjusted for sex, mean income, age > 45 years, and interaction between age and sex [22]. No association with NO_2_ was demonstrated (OR: 1.02; 95% CrI: 0.98 to 1.02) [22].

### 3.4. Juvenile Idiopathic Arthritis

Two North American studies have explored the association between PM_2.5_ and JIA. From a patient population in Utah, 338 cases were identified based on a clinical examination by a rheumatologist. Exposure determination was based on monitoring sites data and no-intercept regression models. RR of 1.60 per 10 *μ*g/m^3^ (95% CI: 1.00 to 2.54) for disease onset was found for children < 5.5 years of age but the results were imprecise when all ages were included in the analysis (RR: 1.11; 95% CI: 0.85–1.45) [23]. The results were not replicated when studying a broader population in America and Canada with systemic-onset JIA [24].

### 3.5. Study Quality

The four studies in RA and two studies in SARDs were all deemed to be of high quality on the Newcastle-Ottawa scale in domains of selection, comparability, and exposure in the case-control studies and domains of selection, comparability, and outcome for the cohort study. Both studies in JIA were rated at lower quality, related to the case-crossover design selected. A summary of the quality assessment is found in Tables 3 and 4.

## 4. Discussion

The goal of our research was to synthesize the published literature on associations between air pollution and the development of rheumatic disease. Air pollution has previously been associated with inflammation and other immune-mediated diseases such as inflammatory bowel disease [2] and multiple sclerosis [3], with the hypothesis built on strong basic science and translational studies [6, 27]. We identified relevant studies in RA, SARDs, and JIA conditions. In a cohort study from the USA and a case-control study from Sweden, no association between an increased RA risk and exposure to NO_2_, SO_2_, or PM was detected. In contrast, the cohort study from Taiwan found increased risk of RA with exposure to higher levels of NO_2_. Surprisingly, the case-control study by De Roos et al. did find an increased risk for RA based on proximity of the primary residence to highways but a potential reduced risk of developing RA in relation to air pollutant exposure [18], which is counterintuitive. In contrast, exposure to PM_2.5_ does appear to confer increased risk for SARDs and was a risk factor for JIA in US children below 5.5 years of age. We additionally identified abstracts on ANCA vasculitis [25] and Kawasaki Disease [26], which reported no association with exposure to PM_10_ and PM_2.5_, respectively, although estimates were not provided.

There are several possible reasons for the observed findings. Just as peak incidence of RA varies with age, there may be periods of life where the impact of air pollutants has greater influence on subsequent susceptibility to developing autoimmune diseases. Just as younger patients appeared to be more vulnerable to an association between air pollutants and JIA onset in Zeft et al.'s study [23], using multivariate analysis controlling for smoking, occupational exposure, home distance to sources of inhaled pollutants, seasonality, and traffic exposure, Orione et al. showed a significant association (odds ratio of 12.2) between carbon monoxide in the third trimester and the subsequent development of juvenile dermatomyositis [28]. Interactions between pollution exposure and specific risk alleles for different autoimmune conditions may also explain the difference in findings of association between air pollutants and different diseases.

Measurement of exposure is another important consideration when interpreting studies of pollution's effects on health. Largely, the studies employed place of residence prior to or at diagnosis to determine exposure, without accounting for places where leisure time, occupation, or daily commute might impact risk, resulting in exposure misclassification [29]. The measurement period, duration, and latency period between subclinical and clinical rheumatic diseases might result in wrongfully attributing exposure to the diagnosis period only. The varied composition of air pollution can make it challenging to overcome the confounding effects of concurrent pollutant exposure. Here distance to roadway studies have been conducted [18, 30], but further information on which pollutants create this heightened risk is required. Yet another consideration proposed is that the range and variability in pollution levels must be sufficiently large to detect associations, which may allow detection of risks limited to higher exposure levels [18].

Our systematic review included a broad search strategy in order to ensure complete identification of relevant articles. We did not perform a meta-analysis because of the small number of eligible studies identified and their heterogeneity. Our systematic review serves as a valuable resource that highlights methodological considerations that should be considered in future research studies that explore the relationship between air pollution and immune-mediated diseases.

## 5. Conclusion

The existing studies suggest evidence for possible associations of PM_2.5_ exposure with SARDs development and JIA in younger age cohorts, but the evidence is less clear for links between air pollutant exposures and the development of RA. Additional epidemiologic work is suggested to improve upon existing analysis methods and expand studies of the effects of air pollution on disease phenotype and prognosis. More basic science and translational studies may also help to discover and explain the mechanisms behind progression from pollution related immune stimulation to the formation of antibodies and ultimately to progression of clinically apparent disease.

## Figures and Tables

**Table 1:** Description of studies included for synthesis.

Disease studied	Author and year	Country or region	Type of study	Sample	Case definition for diagnosis of rheumatic disease	Years of study	Air pollutants studied	Method to determine exposure
Rheumatoid arthritis	Chang et al., 2016 [17]	Taiwan	Cohort	Population at risk NO_2_ exposure, *n* = 247,419, with *n* = 376 cases; PM_2.5_ exposure, *n* = 244,413, with *n* = 236 cases	Administrative data, 1 ICD-9-CM code for RA	2000–2010	NO_2_, PM_2.5_	Monitoring sites
	De Roos et al., 2014 [18]	British Columbia, Canada	Nested case-control	Controls, *n* = 19,066 Cases, *n* = 1,911	Administrative data, 2 ICD-9 codes for RA with minimum 1 visit to physician specialist	1994–2002	NO_2_, SO_2_, PM_2.5_, PM_10_, CO, NO, black carbon, ozone	Land use regression method for black carbon, PM_2.5_, NO_2_, NO Inverse distance weighting method for PM_10_, NO, SO_2_, Ozone, CO
	Hart et al., 2013 [19]	Sweden	Case-control	Controls, *n* = 2,536 Cases, *n* = 1,497	Rheumatologist history and exam	1996–2008	NO_2_, SO_2_, PM_10_	Land use regression
	Hart et al., 2013 [20]	USA	Cohort	Population at risk, *n* = 111,425 Cases, *n* = 858	Self-report and medical chart review	1986–2006	NO_2_, SO_2_, PM_2.5_, PM_10_	Land use regression

Systemic autoimmune rheumatic disease	Bernatsky et al., 2016 [21]	Quebec and Alberta, Canada	Cohort	Quebec estimated population at risk, *n* = 7,977,960 Estimated cases, *n* = 30,330 Alberta estimated population at risk, *n* = 3,053,980 Estimated cases, *n* = 8,180	Administrative data, 2 ICD-9 codes for SARD or 1 ICD-9 code for SARD by a rheumatologist or 1 instance of hospitalization	Quebec, 1996–2011 Alberta, 1993–2007	PM_2.5_	Satellite-derived data of exposure levels at location of residence at time of diagnosis
	Bernatsky et al., 2015 [22]	Calgary, Alberta, Canada	Cohort	Not provided	Administrative data, 2 ICD-9 codes for SARD or 1 ICD-9 code for SARD by a rheumatologist or 1 instance of hospitalization	1993–2007	PM_2.5_, NO_2_	Land use regression

Juvenile idiopathic arthritis	Zeft et al., 2009 [23]	USA	Cohort (case-crossover)	Cases, *n* = 338	Clinical registry	1993–2006	PM_2.5_	Monitoring sites No intercept regression models
	Zeft et al., 2014 [24]	USA and Canada	Cohort (case-crossover)	Not mentioned in abstract	Not specified	Not mentioned in abstract	PM_2.5_	Selected exposure windows but no mention of extrapolation

**Table 2:** Association between air pollutant exposure and the development of rheumatoid arthritis.

Author	Study design	Association reported	Nitrogen dioxide (NO_2_)	Fine particulate matter < 2.5 microns (PM_2.5_)	Fine particulate matter < 10 microns (PM_10_)	Sulfur dioxide (SO_2_)
Chang et al., 2016 [17]	Cohort	HR^*∗*^ per pollutant level^*∗∗*^	Q2, 1.12 (95% CI: 0.83 to 1.52); Q3, 1.53 (95% CI: 1.12 to 2.90); Q4, 1.52 (95% CI: 1.11 to 2.08)	Q2, 1.22 (95% CI: 0.85 to 1.74); Q3, 1.15 (95% CI: 0.82 to 1.62); Q4, 0.79 (95% CI: 0.53 to 1.16)	Not reported	Not reported

De Roos et al., 2014 [18]	Nested case-control	OR per IQR increase^*∗∗∗*^	0.90 (95% CI: 0.85 to 0.96)	0.92 (95% CI: 0.87 to 0.98)	0.91 (95% CI: 0.86–0.96)	0.88 (95% CI: 0.82–0.93)

Hart et al., 2013 [19]	Case-control	OR per IQR increase over average exposure^*∗∗∗∗*^	0.98 (95% CI: 0.90 to 1.07)	Not reported	0.96 (95% CI: 0.88 to 1.04)	1.01 (95% CI: 0.93 to 1.09)

Hart et al., 2013 [20]	Cohort	HR per IQR range increase^*∗∗∗∗∗*^	0.92 (95% CI: 0.85 to 1.00)	0.94 (95% CI: 0.86 to 1.04)	0.92 (95% CI: 0.85 to 0.99)	0.99 (95% CI: 0.90 to 1.09)

HR: hazard ratio; IQR: interquartile range; OR: odds ratio.

^*∗*^Adjusted for age, sex, urbanization level of residence, monthly income, and chronic obstructive pulmonary disease.

^*∗∗*^NO_2_: Quartile 1, <66,213 ppm (referent); Quartile 2, 66,213 to 86,908 ppm; Quartile 3, 86,099 to 99,882 ppm; Quartile 4, >99,992 ppm.

PM_2.5_: Quartile 1, <10,760 *μ*m/m^3^ (referent); Quartile 2, 10,760 to 12,161 *μ*m/m^3^; Quartile 3, 12,162 to 15,056 *μ*m/m^3^; Quartile 4, >15,056 *μ*m/m^3^.

^*∗∗∗*^Adjusted for age, sex, and neighborhood socioeconomic status.

^*∗∗∗∗*^Adjusted for age, sex, smoking status, and educational attainment.

^*∗∗∗∗∗*^Adjusted for age, race, smoking status and pack-years of smoking, age at menarche, parity, duration of lactation, menopause, hormone replacement therapy or oral contraceptive use, physical activity, body mass index, parental occupations, education, marital status, husband's education, family income, and house value.

**Table 3:** Newcastle-Ottawa scale for quality of study assessment: case-control studies.

Case-control studies	Manuscript type	Adequate case definition	Representativeness of cases	Selection of controls	Definition of controls	Comparability of cases and controls	Ascertainment of exposure	Consistency ascertainment	Nonresponse rate	Total
De Roos, Canada, 2014 (RA)	Full	1^*∗*^	1^*∗*^	1^*∗*^	1^*∗*^	2^*∗*^	1^*∗*^	1^*∗*^	1^*∗*^	9^*∗*^
Hart, Sweden, 2013 (RA)	Full	1^*∗*^	1^*∗*^	1^*∗*^	1^*∗*^	2^*∗*^	1^*∗*^	1^*∗*^	1^*∗*^	9^*∗*^
Bernatsky, Alberta and Quebec, 2016 (SARD)	Full	1^*∗*^	1^*∗*^	1^*∗*^	1^*∗*^	2^*∗*^	1^*∗*^	1^*∗*^	1^*∗*^	9^*∗*^
Bernatsky, Calgary, 2015 (SARD)	Full	1^*∗*^	1^*∗*^	1^*∗*^	1^*∗*^	2^*∗*^	1^*∗*^	1^*∗*^	1^*∗*^	9^*∗*^
Zeft, US, 2009 (JIA)	Full	1^*∗*^	1^*∗*^	1^*∗*^	0	1^*∗*^	1^*∗*^	1^*∗*^	1^*∗*^	7^*∗*^
Zeft, US and Canada, 2014 (JIA)	Abstract	1^*∗*^	1^*∗*^	0	0	2^*∗*^	1^*∗*^	0	0	5^*∗*^

For case-control studies, quality was assessed for four domains of selection (case definition, representativeness of cases, selection of controls, and definition of controls), two domains of comparability (study controls for the most important factor and any additional important factor), and three domains of exposure (ascertainment of exposure, same method of ascertainment for cases and controls, and the nonresponse rate). Points are assigned based on specified levels of quality within each domain to a maximum of 9 points.

*∗* denotes the rating system used in the NOS scale.

**Table 4:** Newcastle-Ottawa scale for quality of study assessment: cohort studies.

Cohort studies	Representativeness of exposed cohort	Selection of nonexposed cohort	Exposure ascertainment	Measured outcome not present at study onset	Comparability of cohorts	Outcome ascertainment	Sufficient Follow-up to allow outcome to occur	Adequacy of follow-up	Total
Chang, Taiwan, 2016 (RA)	1^*∗*^	1^*∗*^	1^*∗*^	1^*∗*^	1^*∗*^	1^*∗*^	0	1^*∗*^	8^*∗*^
Hart, USA, 2013 (RA)	1^*∗*^	1^*∗*^	1^*∗*^	1^*∗*^	2^*∗*^	1^*∗*^	1^*∗*^	1^*∗*^	9^*∗*^

For cohort studies, quality is assessed for four domains of selection (representativeness of exposed cohort, selection of the non-exposed cohort, ascertainment of exposure, and demonstration that the outcome of interest was not present at start of study), two domains of comparability (study controls for the most important factor and any additional important factor), and three domains of outcome (method of assessment of outcome, follow-up period, and adequacy of follow-up of cohorts). Points are assigned based on specified levels of quality within each domain to a maximum of 9 points.

*∗* denotes the rating system used in the NOS scale.

## Appendix

### Search Strategy (Medline)

(1) air pollution^*∗*^/or air pollutant^*∗*^/or air polluted/or air contamination^*∗*^/or atmosphere pollution^*∗*^/or atmosphere pollutant^*∗*^/or atmosphere contamination^*∗*^/or atmospheric pollution^*∗*^/or atmospheric pollutant^*∗*^/or atmospheric contamination^*∗*^/or “particulate matter”/or “PM_10_”/or “PM_2.5_”/or ozone/or “O_3_”/or “carbon monoxide”/or carbonmonoxide/or “CO”/or “nitrogen dioxide”/or “NO_2_”/or “sulphur dioxide”/or “sulphur dioxyde”/or “sulfurous anhydride”/or “SO_2_”.ti,ab
(2) Air Pollution/or Particulate Matter/or Ozone/or Carbon Monoxide/or Nitrogen Dioxide/or Sulfur Dioxide.sh.
(3) Or/(1), (2)
(4) Arthritis, Rheumatoid/
(5) rheumatoid arthritis.tw.
(6) exp Lupus Erythematosus, Systemic/
(7) systemic lupus erythematosus.tw.
(8) exp Arthritis, Juvenile/
(9) juvenile idiopathic arthritis.mp. or juvenile arthritis.tw. [mp = title, abstract, original title, name of substance word, subject heading word, keyword heading word, protocol supplementary concept word, rare disease supplementary concept word, unique identifier]
(10) exp Dermatomyositis/or exp Myositis/or exp Polymyositis/
(11) (dermatomyositis or inflammatory myo^*∗*^).mp. or polymyositis.tw. [mp = title, abstract, original title, name of substance word, subject heading word, keyword heading word, protocol supplementary concept word, rare disease supplementary concept word, unique identifier]
(12) exp Scleroderma, Systemic/or exp Rheumatic Diseases/
(13) (SARD or systemic autoimmune rheumatic disease or scleroderma).mp. or systemic sclerosis.tw. [mp = title, abstract, original title, name of substance word, subject heading word, keyword heading word, protocol supplementary concept word, rare disease supplementary concept word, unique identifier]
(14) exp Systemic Vasculitis/or exp Anti-Neutrophil Cytoplasmic Antibody-Associated Vasculitis/or exp Vasculitis/
(15) vasculitis.tw.
(16) (4) or (5) or (6) or (7) or (8) or (9) or (10) or (11) or (12) or (13) or (14) or (15)
(17) (3) and (16).

## List of Abbreviations

RA: Rheumatoid arthritis
JIA: Juvenile idiopathic arthritis
SARDs: Systematic autoimmune rheumatic diseases
PM_2.5_: Particulate matter <2.5 *μ*m in size
PM_10_: Particulate matter <10 *μ*m in size
SO_2_: Sulfur dioxide
NO_2_: Nitrogen dioxide
CO: Carbon monoxide
O_3_: Ozone
SLE: Systemic lupus erythematosus
RR: Relative risk
HR: Hazard ratio
OR: Odds ratio
95% CI: 95% confidence interval
95% CrI: 95% Credible Interval.
